# Case Report: Simil-Appendicitis Presentation May Precede Cardiac Involvement in MIS-C Patient

**DOI:** 10.3389/fped.2022.832391

**Published:** 2022-03-07

**Authors:** Matteo Trevisan, Alessandro Amaddeo, Andrea Taddio, Alessandro Boscarelli, Egidio Barbi, Giorgio Cozzi

**Affiliations:** ^1^Department of Medicine, Surgery, and Health Sciences, University of Trieste, Trieste, Italy; ^2^Institute for Maternal and Child Health Istituto di Ricerca e Cura a Carattere Scientifico (IRCCS) “Burlo Garofolo”, Trieste, Italy

**Keywords:** MIS-C, COVID-19, appendicitis, QT prolongation, myocarditis

## Abstract

**Introduction:**

Multisystem inflammatory syndrome in children (MIS-C) is a new clinical entity characterized by a systemic hyperinflammation triggered by SARS-CoV-2 infection in children and adolescents. This condition could potentially involve all organs with main complications concerning cardiovascular system. Despite up to 90% of patients complain gastrointestinal symptoms (nausea, vomit, and diarrhea), a presentation mimicking acute appendicitis has rarely been reported, and can be the presenting feature of the disease, potentially leading to misdiagnosis and delayed treatment.

**Case Description:**

A 15-year-old boy presented to the Emergency Department for a 2-day history of fever, vomiting, and mild abdominal pain. One month before, the patient complained ageusia and anosmia while his mother tested positive for Sars-CoV2 nasopharyngeal swab. At admission, laboratory tests showed leukocytosis with lymphopenia and elevation of inflammatory markers, while cardiac enzymes, electrocardiogram and echocardiography were unremarkable. An abdominal ultrasound displayed a thickening of terminal ileus and cecum with ascites. Because of the worsening abdominal pain and a physical examination suggestive of acute appendicitis, a laparoscopy was performed but no surgical condition was found. After surgery, fever and generalized malaise persisted, so a cardiac evaluation was repeated, showing a relevant increase in inflammatory markers and cardiac enzymes. Electrocardiogram demonstrated a QTc prolongation with mild decrease in left ventricular ejection fraction at echocardiogram. A MIS-C was diagnosed and intravenous immunoglobulin along with a steroid treatment started. After 36 h, the patient presented a complete clinical recovery with fever cessation. Cardiac anomalies normalized in 3 weeks.

**Conclusion:**

MIS-C has been defined as a systemic inflammation, involving at least two organs, after a previous SARS-CoV2 infection in children and adolescents. Physicians should be aware that while gastrointestinal manifestations are common, a pseudo appendicitis presentation may also occur, leading to misdiagnosis and delayed treatment. This report suggests that in patients with symptoms suggestive of an acute appendicitis, the presence of lymphopenia, hypoalbuminemia and ultrasound images of terminal ileus inflammation, should raise the suspect for MIS-C even without initial overt signs of cardiac involvement.

## Introduction

Multisystem inflammatory syndrome in children (MIS-C), firstly described by Riphagen et al., is characterized by a systemic hyperinflammation triggered by severe acute respiratory syndrome coronavirus 2 (SARS-CoV-2) infection in children and adolescents (1, 2). According to the Centre of Disease Control and Prevention (CDC), case definition of MIS-C includes age of <21 years, fever for at least 24 h, elevation of inflammatory markers, serious illness leading to hospitalization or at least two organs involvement (cardiac, renal, respiratory, hematological, gastrointestinal, dermatological, or neurological) with a history of possible SARS-CoV2 infection (positive real time-polymerase chain reaction, positive serology or contact with COVID-19 in the past 4 weeks). Usually developed after 4-6 weeks from primary infection, MIS-C is the most dangerous complication of SARS-CoV2 infection in children (2, 3).

While adult patients with COVID-19 present gastrointestinal symptoms in only 15% of cases, up to 90% of MIS-C patients complain abdominal pain, diarrhea and vomiting. Gastrointestinal symptoms may be the first symptoms in MIS-C patients mimicking other conditions such as gastrointestinal infections or inflammatory bowel diseases (2, 4–7). For this reason, laboratory exams and abdominal ultrasound can be helpful in differential diagnosis, though at the onset they can be indeterminate or unremarkable (2, 4, 6–8).

Cardiovascular involvement is present in up to 80% of MIS-C patients, usually arising after 6-8 days of fever, with cardiogenic shock as its most life-threatening manifestation (2, 9). Due to a high prevalence of intensive care needs, directly associated to the elevation of myocardial and inflammatory markers, a prompt recognition and treatment of MIS-C patient is mandatory (10). Up to now, immunomodulant treatment seems effective to recover from cardiac damage, but no studies evaluated long-term cardiovascular sequalae (2, 9, 11, 12).

Here, we report a case of MIS-C in an adolescent boy with pseudo-appendicitis symptoms followed by myocarditis and heart conduction abnormalities.

## Case Description

We report the case of a 15-year-old adolescent who presented to the pediatric emergency department with a 2-day history of fever, vomiting and diarrhea and mild abdominal pain. His history was remarkable for a period of anosmia and ageusia experienced 1 month before presentation. In that occasion, two nasopharyngeal swabs for SARS-CoV-2 tested negative, while his mother's one tested positive.

At admission, he was febrile and reported a severe asthenia. Vital signs were normal, except for mild tachycardia (hearth rate 140 beats/min) and fever of 39°C. Capillary refill time was lower than 2 s. The cardio-thoracic examination was unremarkable, while a mild diffuse tenderness on abdominal palpation was elicited. No rashes or cutaneous lesions were noted. Laboratory tests showed mild leukocytosis (white blood cells 10,480 mm^3^), with lymphopenia (550 mm^3^), elevation of C-reactive protein (CRP 137 mg/L, normal value <5 mg/L), mild elevation of D-dimer (1,249 ng/ml; n.v. <500 ng/ml) and fibrinogen within the normal values (430 mg/dl, n.v. 174-434 mg/dl). A nasopharyngeal swab to check the presence of SARS-CoV2 tested negative. Considering the history of recent ageusia and anosmia, the presence of fever, asthenia and gastrointestinal symptoms within elevation of inflammatory markers and lymphopenia, a diagnosis of MIS-C was suspected.

No signs of cardiac involvement were noted: myocardial markers were in normal range (cardiac troponin 2 ng/L [n.v. <19 ng/L] and brain natriuretic peptide BNP 200 pg/ml [n.v. <300 pg/ml]) with normal electrocardiogram and echocardiography. Nevertheless, the absence of cardiac involvement did not preclude a MIS-C diagnosis.

**Figure 1:**
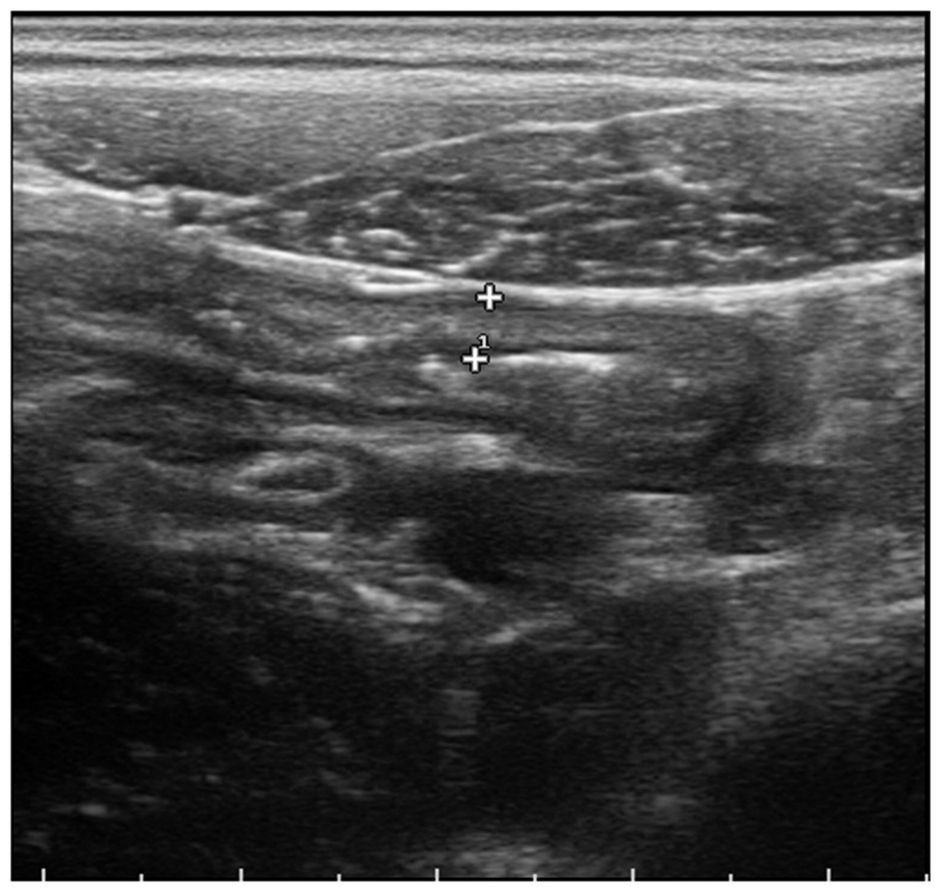
Abdominal ultrasound showing transmural thickening of the terminal ileus.

Within 24 h from admission the patient rapidly worsened, developing a progressive abdominal pain in the right lower quadrant, with local guarding and rebound tenderness. Abdominal ultrasound showed a thickening of the terminal ileus with ascites and mesenteric lymphadenopathy, while the appendix was not detected (Figure 1).

A laparoscopic exploration was performed to rule out acute appendicitis. The ileus and cecum appeared thickened and inflamed, while the appendix was normal. Broad spectrum antibiotic treatment was started.

**Figure 2:**
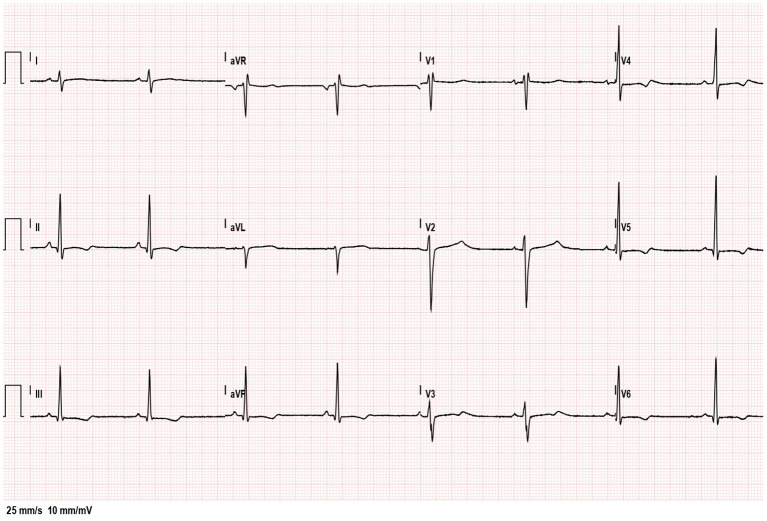
Electrocardiogram showing sinus bradycardia (HR 50 beats/min) with QT tract prolongation of 494 ms, diffuse T-waves alteration and minimal right branch block.

Four days after admission and 2 days after surgery, despite antibiotic therapy the patient was still febrile and markedly asthenic. Thus, a second cardiological evaluation was performed, showing increased inflammatory and myocardial markers (CRP 250 mg/L, cardiac troponin 65 ng/L, BNP 9,195 pg/ml), negative T waves along with prolonged QT interval (490 ms) at ECG (Figure 2) and a reduced left ventricular ejection fraction (LVEF 55%) with a tricuspid regurgitation at echocardiography. According to the simultaneous presence of cardiac and abdominal involvement, a diagnosis of MIS-C was made and treatment with intravenous immunoglobulins (2 g/kg) and steroids (methylprednisolone 2 mg/kg) was started. Due to the concomitant myocarditis, he received a prophylactic anticoagulation (enoxaparin 4,000 IU/day) and antiplatelet therapy (acetylsalicylic acid 100 mg/day). After 24 h the patient had a prompt recovery with cessation of fever, abdominal pain and malaise. In few days inflammatory and cardiac markers progressively decreased to normal values, while ECGs and echocardiogram normalized in 3 weeks. The patient was kept in steroids for 1 month with a gradual reduction of the doses. Exercise restriction was recommended for 6 months, when the patient will undergo cardiac magnetic resonance imaging (MRI).

## Discussion and Conclusion

Here, we reported the case of an adolescent with MIS-C in which gastrointestinal symptoms resembled an acute appendicitis. The rapid worsening of abdominal symptoms with simil-appendicitis presentation led to an unnecessary explorative laparoscopy and treatment delay. The subsequent cardiovascular involvement and the increasing inflammatory markers allowed the right diagnosis and treatment with prompt and complete recovery. Undoubtedly, MIS-C diagnosis was yet suspected at the onset of the disease due to the two organs involvement (abdominal and hematological systems), the fever and elevation of inflammatory markers. However, the progressive worsening of abdominal pain led to consider a surgical condition.

Despite gastrointestinal symptoms (abdominal pain, emesis and diarrhea) are common features in MIS-C patients, only in a few cases these manifestations resemble an acute appendicitis (5–7, 13–15). Moreover, when these manifestations precede other organ involvement a differential diagnosis between inflammatory bowel disease, abdominal surgical conditions or severe infections could be difficult (13–16). Typical laboratory findings in MIS-C patients are lymphopenia with neutrophilia, increased PT and D-dimer, hypoalbuminemia, hypertransaminasemia, elevation of inflammatory (CRP, ESR, ferritin and fibrinogen) and cardiac (troponin and BNP) markers (2). As in our case, among all these values, increased inflammatory markers, lymphopenia and hypoalbuminemia are common findings in patients with gastrointestinal symptoms (4, 6). Reviewing abdominal imaging in MIS-C patients, common US findings are ascites, acalculous cholecystitis, bowel wall thickening and mesenteric adenitis (4, 6–8). Moreover, in a systematic review Rouva et al. reported an incidence of 30% of acute abdomen among MIS-C patient with gastrointestinal symptoms. The final diagnoses were mostly non-surgical (76.4%) and, as in our case, in around 25% of cases a terminal ileitis/ileocolitis was found (8). Nevertheless, few authors even described MIS-C patients with acute abdomen presentation, in some cases simultaneous with cardiac involvement, for whom symptoms and ultrasound imaging were misleading. Lastly, a diagnosis of MIS-C should not preclude a possible diagnosis of appendicitis. Indeed, in some cases of acute abdomen in MIS-C, appendicitis was confirmed at macroscopic and histological evaluation (17). Moreover, few authors described a direct involvement of appendix in MIS-C, where the systemic inflammation led to vasculitis, thrombus formation and ischemic necrosis of the intestinal wall (18). Therefore, in the first week of disease, gastrointestinal symptoms, laboratory exams and abdominal US could be misleading, especially if no other organ involvement is already present. Although cardiac involvement is not mandatory for MIS-C diagnosis, the absence of laboratory or functional markers of cardiac damage along with the quick development of acute abdomen in the first hours, led to an early misdiagnosis and delayed treatment. However, we cannot assume that an early treatment with steroid and IVIG would have avoided a cardiac involvement. Indeed, therapeutic strategy in MIS-C are driven to its similarity with Kawasaki disease.

In our case, retrospectively, the persistent asthenia could be seen as an early marker of cardiac involvement, a well-described symptom in MIS-C patient with cardiac manifestations (12). Nevertheless, no elevation of cardiac enzymes or decreased ventricular function were noted in the patient until the second week of disease.

In regard to the risk of intensive care admission and inotropic support in MIS-C patient, a prompt and right diagnosis is mandatory. Up to 80% of patients develop cardiovascular involvement ranging from only mild elevation of cardiac markers (troponin and pro-BNP) to cardiogenic shock. If MIS-C shares some Kawasaki's disease features, the former usually results in more severe ventricular dysfunction and myocarditis. Cardiogenic shock has been proposed to be the result of either myocardial viral damage or “cytokine storm” vasodilatation. Other cardiovascular complications encompass coronary artery aneurism/dilatation and conduction abnormalities. According to a review of cardiac involvement in MIS-C, coronary artery dilatation is reported in up to 25% of patients, while heart conduction abnormalities showed a 7-60% prevalence. Typical arrhythmias are first-degree atrioventricular block, QTc prolongation and ST segment changes. The American Heart Association suggests repeated ECG during the acute phase, but telemetry is needed if any arrythmias occur. From several large studies, early immunomodulatory treatment seems to resolve cardiac damage in most of all cases, but little is known about cardiac sequelae in MIS-C patients. Once myocardial damage occurs, an expert consensus recommends exercise restriction for 6 months with a cardiac MRI at 3 or 6 months to evaluate heart function.

Up to now, no randomized trials have been developed for treatment and management of MIS-C, but due to the similarities between Kawasaki disease and MIS-C, an immunomodulatory approach with IVIG and steroids is recommended. Moreover, antiplatelet treatment and prophylactic anticoagulation are suggested once myocardial or coronary involvement are present. As in our case, several retrospective studies reported, in the majority of patients, normalization of myocardial markers, ECGs abnormalities and ventricular dysfunction after immunomodulatory treatment (9, 12). Nevertheless, longer studies and trials are needed to evaluate treatments and chronic sequalae.

In conclusion, our case highlights how gastrointestinal involvement in MIS-C could mimic acute appendicitis, and this presentation may precede cardiac involvement, leading to possible misdiagnosis and delayed treatment.

In patients with a recent exposure to SARS-CoV-2 a clinical presentation with fever, asthenia, and gastrointestinal symptoms should be seen as highly suggestive of MIS-C. This suspect may be supported by the presence of an increased inflammatory markers, lymphopenia and hypoalbuminemia and images suggestive of terminal ileitis on ultrasound. Even in the absence cardiac involvement at presentation the diagnosis of MIS-C should not be ruled-out and a strict cardiological follow-up should be performed.

## Data Availability Statement

The raw data supporting the conclusions of this article will be made available by the authors, without undue reservation.

## Ethics Statement

Written informed consent was obtained from the individual(s), and minor(s)' legal guardian/next of kin, for the publication of any potentially identifiable images or data included in this article.

## Author Contributions

MT, GC, and AA wrote the first draft of the manuscript. GC, AA, AT, AB, and EB reviewed and revised the final manuscript. All authors followed the patient clinically. All authors listed on the manuscript have seen and approved the final version of the manuscript.

## Conflict of Interest

The authors declare that the research was conducted in the absence of any commercial or financial relationships that could be construed as a potential conflict of interest.

## Publisher's Note

All claims expressed in this article are solely those of the authors and do not necessarily represent those of their affiliated organizations, or those of the publisher, the editors and the reviewers. Any product that may be evaluated in this article, or claim that may be made by its manufacturer, is not guaranteed or endorsed by the publisher.

## Abbreviations

BNP: Brain natriuretic peptide
CDC: Center for disease control and prevention
CRP: C-reactive protein
ECG: Electrocardiogram
ESR: Erythrocyte sedimentation rate
IVIG: Intravenous immunoglobulin
LFEV: Left ventricular ejection fraction
MIS-C: Multisystem inflammatory syndrome in children
MRI: Magnetic resonance imaging
N.V.: Normal value
US: ultrasound.
